# No Association of COMT (Val158Met) Genotype with Brain Structure Differences between Men and Women

**DOI:** 10.1371/journal.pone.0033964

**Published:** 2012-03-30

**Authors:** Anna Barnes, Matti Isohanni, Jennifer H. Barnett, Olli Pietiläinen, Juha Veijola, Jouko Miettunen, Tiina Paunio, Päivikki Tanskanen, Khanum Ridler, John Suckling, Edward T. Bullmore, Graham K. Murray, Peter B. Jones

**Affiliations:** 1 Department of Psychiatry, University of Cambridge, Addenbrooke's Hospital, Cambridge, United Kingdom; 2 Brain Mapping Unit, University of Cambridge, Cambridge, United Kingdom; 3 Department of Psychiatry, Institute of Clinical Medicine, University of Oulu and Oulu University Hospital, Oulu, Finland; 4 Department of Public Health Science and General Practice, Institute of Health Sciences, University of Oulu, Oulu, Finland; 5 Cambridge Cognition, Cambridge, United Kingdom; 6 Institute for Molecular Medicine Finland FIMM, University of Helsinki, Helsinki, Finland; 7 National Institute for Health and Welfare, Helsinki, Finland; 8 Department of Psychiatry, University of Helsinki, Helsinki, Finland; 9 Department of Radiology, Institute of Diagnostics, University of Oulu, Oulu, Finland; 10 Behavioural and Clinical Neuroscience Institute, University of Cambridge, Cambridge, United Kingdom; Beijing Normal University, Beijing, China

## Abstract

We examined the effect of the catechol-O-methyltransferase (COMT) Val158Met polymorphism (rs4680), on brain structure in a subset (N = 82) of general population members of the Northern Finland 1966 Birth Cohort, selected through a randomization procedure, aged 33–35. Optimised voxel-based morphometry was used to produce grey matter maps from each subject's high resolution T1 weighted brain magnetic resonance images, which were subsequently entered into a general linear model with COMT genotype as defined by Met allele loading, gender and genotype by gender interaction as independent variables. Additional analyses were carried out on grey matter volumes within the dorsal lateral pre-frontal cortex (DLPFC) to examine effects on overall DLPFC volume and also using the DLPFC as a mask for voxelwise analyses, as this is an area previously reported as associated with Met allele loading. We failed to find any statistically significant association with grey matter volume and Met allele loading in the COMT gene or interaction affects between COMT and gender in either the whole brain voxel-wise analysis or in the area of the DLPFC.

## Introduction

Catechol-O-methyltransferase (COMT) catalyzes the first step in a major degradation pathway of the catecholamine neurotransmitters: noradrenaline, adrenaline and dopamine. The COMT gene, located on chromosome 22q11, contains a functional polymorphism (Val158Met; rs4680), with Met alleles resulting in a fourfold decrease in enzyme activity compared to Val homozygotes at body temperature [1]. This decrease in activity leads to slower inactivation of released dopamine within the brain, notably in the prefrontal cortex where COMT may be responsible for around half of dopamine decline [2]. The polymorphism is common in the general population and may have small effects on cognitive functions that rely on the prefrontal cortex, primarily those involving executive function [3], [4]. COMT's effects seem larger and more robust however at the level of brain activation: functional neuroimaging studies consistently show increased prefrontal activation (implying reduced efficiency) in Val allele carriers compared with Met carriers while completing tests of executive function [5].

The majority of previous studies of the effect of variants at the COMT Val158Met polymorphism have concentrated on functional imaging and cognitive performance, perhaps because dopamine is known to play a critical role in executive function [5], [6]. Since this key neuromodulatory transmitter plays a critical role in synaptic plasticity and learning, then it would be expected that it may also be important for brain structure (at least at the level of the synapse). For example, dopamine can mediate synaptic plasticity through multiple mechanisms [7]. Firstly, it controls the activity of AMPA and NMDA receptors through phosphorylation, secondly it regulates voltage-gated ion channels such as sodium and calcium channels by affecting phosphorylation state, and thirdly it regulates gene expression (through phosphorylation of specific transcription factors).

Dopamine does play a role in embryological development, as there is evidence that dopamine depletion in drosophila lead to lethality, developmental retardation, and abnormal ovarian development [8]. Studies in genetically modified dopamine deficient mice indicate that such mice have grossly normal brain structure, but with subtle abnormalities such as reduced total brain volume. Moreover, studies in both experimental animals and humans show that dopamine receptor antagonism can have complex effects on brain structure. For example, Dorph-Petersen and colleagues [9] documented that monkeys treated with olanzapine or haloperidol (both dopamine receptor antagonist agents) showed decreases in grey matter volume in all major brain regions. Whereas imaging studies in schizophrenia patients have demonstrated increases in grey matter in sub-cortical regions after several weeks of anti-psychotic medication [10], [11]. These results would seem to indicate that dopamine, and dopamine receptor blockade, modulate brain structure in a complex fashion.

Evidence from twin studies suggests that brain structure is heritable, although the specific contribution of particular genetic variants in determining brain structure is unclear [12], [13], [14]. Previous authors have hypothesized that variation at Val158Met could be associated with separable aspects of adult brain structure, and have tested this hypothesis using magnetic resonance imaging in both healthy people and patients with mental disorders. Interestingly, in one of the previous reports to examine whole brain, voxel-wise structure and COMT genotype in healthy volunteers, Zinkstok and colleagues failed to find any association with brain structure and genotype but found genetic effects on age-related differences in grey and white matter density in women but not in men [15]. Kates and colleagues also found gender specific effects when they studied 52 children with velo-cardio-facial syndrome: a condition where individuals have a deletion of more than 40 genes on chromosome 22 in a region including the COMT gene, leaving them with only one copy of that gene [16]. That study also showed a sex x genotype interaction, with boys carrying a Val allele having greater dorsal prefrontal grey matter than boys with a Met allele.

There is an emerging body of evidence that COMT has sexually-dimorphic effects on a range of neurobiological phenotypes [17]. Functional studies have demonstrated that COMT activity in human post-mortem PFC tissue is significantly higher in men than women [18], and in COMT knockout mice, frontal cortical dopamine levels are affected in males but not females [19]. The effects of the Val158Met polymorphism also appear to be sexually dimorphic in humans. The largest studies of cognition suggest that Val158Met affects executive function only in men [20], and case-control meta-analyses find significant associations between Val158Met and panic disorder in women but not men, and significant association with obsessive-compulsive disorder in men but not women [21]. For COMT, the likely explanation for sex-specific associations, are the bilateral relationships between COMT and oestrogen-related compounds. Specifically, oestrogens mediate COMT expression [22] and COMT metabolizes catechol oestrogens, a process which is itself regulated by Val158Met variation [23].

This study aimed to clarify associations between Val158Met and whole-brain and regional volumetric differences in a group of subjects sampled randomly from a population based birth cohort in Northern Finland, a region known to have a high degree of genetic homogeneity. We hypothesized that genetic variation at the COMT Val158Met SNP would be associated with subtle brain morphological deficits, with dose-dependent associations between morphological variation and Met allele dosage. In particular, given the mounting body of evidence that COMT's effects are sexually dimorphic, we predicted that there would be significant sex x genotype interactions on morphological variation.

## Methods

### Ethics Statement

Permission to gather data was obtained from the Ministry of Social and Health Affairs and the study design has been approved by and is under the review of the Ethical Committee of the Northern Ostrobothnia Hospital District

### Subjects

The Northern Finland 1966 birth cohort (NFBC66) is an unselected, general population birth cohort ascertained during mid-pregnancy (n = 12,068). The cohort represents 96% of the live born children in the Finnish provinces of Lapland and Oulu with an expected delivery date during 1966 [24]. Between 1999 and 2001, when the cohort members were between 33 and 35 years old, MRI data were collected on 104 cohort members randomly sampled from the Oulu region. The sampling was truly randomised in a gender stratified manner (as one motivation for recruiting this sample was to serve as a general population comparison group for a psychiatric group with a higher incidence in men). MRI data and genetic information at Val158Met were available on 82 cohort members (48 men). Details of all subjects that underwent MR scanning for the purposes of the North Finland 1966 Birth Cohort field study are published by Tanskanen et al [25]. Educational level reached by the end of 1997 was collected from Statistics Finland and was categorised into basic (9 years or less), or secondary (10 to 12 years), and tertiary level (over 12 years). Proxy IQ scores were created by taking the average score for each subject from a battery of cognitive tests after normalizing the individual scores to the sample mean within each cognitive test scaled to 100 [26] and reported for each genotype. Details listed in table 1.

**Table 1:** Demographics.

	VVMean (95% C.I)	VMMean (95% C.I)	MMMean (95% C.I)
**Gender** (N = 82)	M = 7, F = 9	M = 29, F = 20	M = 12, F = 5
**Handedness** (N = 82)	R = 15, L = 1	R = 47, L = 2	R = 15, L = 2
**Education** (N = 82)			
Basic (>9 yrs)	0	3	0
Secondary (10–12 yrs)	10	28	14
Tertiary (12+yrs)	6	17	4
**Proxy IQ** (N = 73)	100.36±0.98	99.8±0.98	100.4±0.79

List of demographics for the 82 subjects included in this study, grouped by genotype. Data on educational level reached by the end of 1997 was collected from register of the Statistics Finland. Handedness data obtained at the time of MR scan.

This selected imaging cohort had no history of psychosis according to the Finnish Hospital Discharge Register.

### Genotyping

82 subjects (48 men) from the neuroimaging study provided a blood sample, from which DNA was extracted according to standard laboratory protocols. These samples were genotyped for the COMT Val158Met polymorphism. Sequenom's homogenous Mass Extend (hME) MassARRAY technology (Sequenom, San Diego, CA, USA) was utilized for genotyping. The PCR and extension primers were designed using SpectroDESIGNER version 2.0. The quality of genotyping was ensured by including eight water controls, and eight duplicated DNA samples on every plate. PCRs were performed in a total reaction volume of 5 ml using 7.5 ng of genomic DNA. The alleles were automatically called by Sequenom's MassARRAY Typer software and verified by two independent reviewers.

### Image acquisition and processing

Structural MRI data were acquired from all participants on a GE Signa system (General Electric, Milwaukee, WI) operating at 1.5T, Oulu University Hospital, Finland. T1 weighted SPGR images of the whole brain were collected; slice thickness 1.5 mm and in plane voxel size 0.94×0.94 mm, TR = 35 ms, TE = 5 ms, Flip Angle = 35°. The images were quality controlled by radiological screening.

Grey matter volume maps were constructed for each subject's image using the current version of FSLVBM (v1.1) (http://www.fmrib.ox.ac.uk/fsl/fslvbm/index.html). First, structural images were brain-extracted using the brain extraction tool (BET) [27] with the additional option of removing slices that included excessive data below the cerebellum. Next, tissue-type segmentation was carried out using FAST4 [28]. The resulting grey-matter partial volume images were then aligned to MNI152 standard space using the affine registration tool FLIRT [29], [30], followed by a nonlinear registration using FNIRT [31], [32] which uses a b-spline representation of the registration warp field [33]. The resulting images were averaged to create a study-specific template (equal numbers from each genotype and sex), to which the native grey matter images were then non-linearly re-registered. The registered partial volume images were then modulated (to correct for local expansion or contraction) by dividing by the Jacobian of the warp field. Total grey matter volume before spatial normalisation for each subject was then calculated using fslstats (http://www.fmrib.ox.ac.uk/fsl/avwutils/index.html). The modulated segmented images were then smoothed with an isotropic Gaussian kernel with a sigma of 2 to minimise slight misregistration errors.

### Statistical analysis

Total grey matter volumes were analysed statistically using SPSSv16.0.2: (http://www.spss.com/software/statistics/). Smoothed grey matter maps were statistically analysed using permutations methods in FSL. Associations between genotype and grey matter maps were analyzed using a linear regression model at each voxel using permutation-based methods implemented in FSL software package (http://fsl.fmrib.ox.ac.uk/fsl/randomise/). Statistical inference using permutation based statistics within FSL is based on Threshold-Free Cluster Enhancement (TFCE) and is a method for finding “clusters” within MRI data without having to specify a single peak threshold above which groups of voxels are defined as a cluster. All analyses were performed using 5000 permutations and the results are reported at a p<0.05 significance level after correction for multiple comparisons.

Associations between COMT genotype and adult brain structure (total grey matter volume and voxel-wise grey matter volume) were tested using the general linear model with the following independent variables: COMT genotype as a linear ordered variable (VV = 1, MV = 2, MM = 3), gender (male, female), and an interaction term of genotype by gender. Post-hoc voxel-wise linear regressions of COMT genotype with grey matter volume (GMV) for each gender separately were also performed on both total GMV (TGMV) and voxel-wise GMV.

For the purposes of voxelwise analysis the relationship between grey matter and COMT genotype was estimated by fitting the following linear regression model within a permutations framework at each intracerebral voxel in standard space:
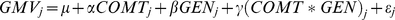
(1)


Here GMV*_j_* denotes the volume of grey matter estimated at a given voxel for the *j*th individual; μ is the overall mean; α is the coefficient of association between grey matter (structure) and COMT genotype at a voxel; β is the coefficient of association between grey matter and sex and γ is the coefficient of association of the interaction term of genotype and sex and grey matter volume and ε*_j_* is the random variation. The independent variable COMT*_j_* denotes genotype of the *j*th individual. GEN*_j_* denotes the gender of the *j*th individual.

We also employed a simpler model to examine the effect of COMT on GMV without testing for a COMT*gender interaction:
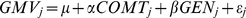
(2)


### Region of interest analysis

All voxel-based analyses were initially performed at the whole-brain level. Additionally, because of previous evidence linking COMT to the prefrontal cortex, and in order to increase statistical power, we repeated our voxel-wise analyses within the dorso-lateral prefrontal cortex using a small volume correction. [34]. Our DLPFC mask was created following the method of Honea and colleagues, using with the WFU PickAtlas (http://www.fmri.wfubmc.edu; Advanced Neuroscience Imaging Research Core, Wake Forest University, Winston-Salem, NC, USA) by selecting Brodmann areas 9, 10, 45 and 46 with a dilation of 1 mm. Finally we extracted the total GMV for the DLPFC ROI for each individual and repeated our analyses on total DLPFC GMV.

### Power calculation

We performed a power calculation in order to quantify our power to detect an effect of genotype on DLPFC volume. With this number of subjects (n = 82), we calculated that we would be able to detect a linear effect of COMT genotype (number of Met alleles) explaining 10% of the variance (R^2^ = 10%, a medium effect size) in DLPFC volume at p = 0.05 with 85% statistical power (http://danielsoper.com/statcalc3 - statistical power calculator for multiple regression).

## Results

### Global effects of genotype on brain volume

The analysis of total grey matter volume before spatial standardisation yielded the following results: group means and standard deviations for TGMV for COMT genotype were; VV(N = 16) = 658.81 ml±60.03 ml, VM (N = 48) = 636.36 ml±55.15 ml, MM (N = 18) = 667.11 ml±45.25 ml (p = 0.085, F = 2.5, df = 2) and for men and women; men (N = 48) = 667.82 ml±50.72 ml, women (N = 34) = 618.79 ml±48.62 ml (p = 0.000035, t = 4.4, df = 80). The overall regression model including genotype, sex and a genotype x sex interaction as factors was significant at p = 0.001, F = 6.44, df = 3, however none of the main effects of genotype (beta = −8.598, 95% CI −35.8 to 18.7, p = 0.532), sex (beta = 27.144, 95% CI −47.0 to 101.3, p = 0.468) or the genotype by sex interaction term (beta = 11.281, 95% CI −24.2 to 46.8, p = 0.530) were significant. In the simpler model without the interaction term, there was a highly significant effect of gender (beta = 49.5, 95% CI 26.7 to 72.3, p = 0.00004), but no significant effect of genotype on TGMV (beta = −1.973, 95% CI −19.4 to 15.4, p = 0.822).

### Regionally specific effects of genotype on brain volume

The FSL randomisation analysis of the same overall regression model showed no significant linear relationship of GMV with met allele loading at any location in the GM maps at a p-level<0.05, TFCE, corrected for multiple comparisons. Nor was there an interaction effect between gender and genotype (although gender itself was associated in GMV differences in widespread cortical and subcortical regions). The null results were the same for the small volume corrected voxelwise analysis using a region of interest defining the DLPFC. We also extracted the total volume of our DLPFC ROI for each subject (Table 2) and subjected that to the same analysis: there was no significant effect of genotype (beta = 0.846, 95% CI −1.5 to 3.2, p = 0.485) or gender by genotype interaction (beta = −0.767, 95% CI −3.9 to 2.3, p = 0.627). In a simpler model with no interaction term there was an effect of gender (beta = −4.65, 95% CI −6.8 to −2.4, p = 0.00008) but no effect of genotype (beta = 0.395, 95% CI −1.1 to 1.9, p = 0.61).

**Table 2:** DLPFC volume according to genotype.

	VVMean (95% C.I)	VMMean (95% C.I)	MMMean (95% C.I)
**DLPFC volume (mls)**	80.0 (77.7 to 82.5)	78.7 (77.2 to 80.2)	80.0 (77.3 to 82.6)
**Men: DLPFC volume (mls)**	79.0 (75.2 to 82.8)	77.9 (75.6 to 79.9)	79.0 (76.3 to 81.7)
**Women: DLPFC volume (mls)**	80.9 (77.2 to 84.6)	79.9 (77.4 to 82.3)	82.4 (73.7 to 91.0)

Dorsolateral prefrontal cortex (DLPFC) volume according to genotype at COMT Val158Met in men and women.

### Additional analyses

We repeated our voxel-based analyses with a more lenient statistical correction threshold (p = 0.01 uncorrected), but we did not find any significant interaction effect, nor any significant effect of genotype when dropping the interaction term. In all previous analyses we had included genotype as a linear term based on number of Met alleles; we repeated our genotype analyses to see if there were brain structural differences between Val carriers versus Met/Met individuals or between Met carriers and Val/Val individuals, but there were no significant differences.

## Discussion

In healthy individuals from an ethnically-homogenous, general population based cohort, the COMT Val158Met polymorphism was not associated with total or voxel-wise GM volume nor was there an interaction between genotype and sex. A further small volume corrected voxel-wise analysis using region of interest analysis based on the DLPFC, previously reported as having a trend-level association with the COMT polymorphism [34] also failed to show a significant relationship.

Issues concerning sample size and statistical power are an important factor to consider here: we detected no effect, but it is possible that future larger studies may be able to show smaller but true effect size. Our power analysis suggested we had good power to detect a small effect size of a linear association between genotype and overall DLPFC volume. Power calculation in voxel-wise analyses are more complex, but two studies by Suckling et al [35], [36] exploring the power of magnetic resonance imaging trials to detect the presence of voxel-wise brain structure differences associated with pathology, demonstrate that with a simple two sample t-test (N = 26 in each group) at a level of p = 0.05 with 80% power, the minimum detectable difference in grey matter volume in much of the frontal cortex is ∼3%, with larger differences in GMV needed to detect a significant group difference in other brain regions. In the context of these results the numbers in our sample are acceptable for that required to detect voxel-wise changes comparing Val carriers with Met/Met individuals (or comparing Met carriers with Val/Val individuals). Although our power to detect interaction effects is less than our power to detect main effects, inspection of the mean DLPFC volumes in men and women (Table 2) show no suggestion of interaction effects. Indeed there is no evidence of a linear association between number of Met alleles and DLPFC volume in either sex, as in both sexes the heterozygotes had slightly lower DLPFC volumes than the homozygotes. It could be that the subtle and distributed nature of the effects of this polymorphism is not best suited for investigation with conventional GLM statistics or this type of brain structure metric. It is possible that studies investigating the topographical properties of grey matter structure such as cortical gyrification or the use of multivariate imaging statistics may be more sensitive methods for genetic imaging studies.

### Relationship to previous studies in healthy populations or mixed case-control studies

To date, the study by Zinkstok and colleagues in 154 young adults is the largest brain morphology COMT and grey matter density (not volume) study in healthy adult volunteers; they showed no effect of COMT genotype on brain structure in either men or women, and no effect of gender by genotype interaction [15]. However, they did find an age related association of GM volume to Val alleles in women only using GM density not volume images. Honea and colleagues [34] studied associations between COMT genotype and GMV in 151 healthy adults; amongst other analyses, the authors used a region of interest approach to examine dorsolateral prefrontal cortex (DLPFC) volume and showed that there was a trend for volume reductions in Met carriers within part of the DLPFC. Cerasa and colleagues measured a differential effect of COMT genotype on frontal lobe volume and hippocampal volume in 57 volunteers, with Val alleles being associated with increased frontal volume and Met alleles associated with increased hippocampal volume [37]. These studies showing trend level results, or effects of COMT in interaction with other factors in complex fashion, or differential effects of Met alleles in different parts of the brain, need to be considered in the light of studies that found no effect of COMT genotype on brain structure. Ho and colleagues found no effect of COMT genotype on frontal lobe volume in 84 controls [38]. Ohnishi and colleagues examined COMT Val158Met in relation to brain structure in a sample of 76 healthy controls and 47 schizophrenia patients [39]. Although they found effects of genotype within patients, they found no effect in controls, even at a low statistical threshold of p<0.05 uncorrected. In a sample of 61 controls, Dutt and colleagues found no significant effects of genetic variation in COMT Val158Met on hippocampal or lateral ventricular volume [40]. Two recent large studies have examined effects of variation at COMT genotype and cortical thickness in large samples; Cerasa et al in 149 adults [41], and Shaw et al [42] in 206 children and adolescents showed that met alleles were associated with increased cortical thickness in partially overlapping fronto-temporal regions.

### Relationship to previous studies in patient samples

Several studies examining COMT genotype variation and association with brain structure in patient samples have been negative; these include no effect of COMT genotype on hippocampal or lateral ventricle volume a sample of over 300 psychosis patients and relatives [40], and no effect of COMT genotype on frontal volumes in 159 schizophrenia patients [38]. However, there have been positive associations in some groups [39], [42]. McIntosh and colleagues [43] found a negative effect of Val alleles on anterior cingulate density in a population of individuals at high genetic risk for schizophrenia. Kates and colleagues found that the Val allele was associated with greater dorsal prefrontal grey matter volume than the Met allele in boys with velo-cardio-facial syndrome; the reverse effect was seen in girls [16].

### Understanding the variability in existing studies of associations between COMT genotype and brain structure

Reviewing the existing literature, it appears that some studies have found no effect of COMT genotype on brain structure [38], [40]; some have found that Met alleles are associated with increased measures of volume/density/thickness in frontal or temporal regions [37], [41], [42] and some have found that Met alleles are associated with decreased measures of volumes or density in frontal or temporal regions [34], [37]. The studies that have found associations have sometimes found associations in patient populations but not in controls and sometimes found associations of COMT genotype interacting with some other variable, whether it be psychiatric disorder, gender or complex age and gender interactions [15], [16], [39]. Honea and colleagues [34] identify a number of factors that could explain this degree of variability in results; differences in methodology, study populations, statistical thresholding and gene-gene or within gene SNP-SNP interactions may all contribute. For example, in a study of 171 individuals, there were small localised GM reductions in bilateral caudate and ventral prefrontal cortex in subjects who were homozygous for the Val allele and were also carriers of the A allele of rs1130233 in the AKT1 gene, indicating the potential importance of studying the effect of gene-gene interactions on brain structure [44]. An additional possibility is that there is no effect of genetic variation at COMT Val158Met on brain structure, and that the heterogeneity of previous findings in opposing directions of effect simply reflect random variations about a null effect, and thus represent type I error. If there is an effect of COMT genotype on brain structure, it appears to be of a complex nature.

Our study has the advantage that it was nested within a population-based birth cohort, comprised of subjects invited to participate through a randomisation procedure, which is likely to result in a more representative sample than many comparable studies, however, we do realise that this may mean that it is not representative of other populations. We used an optimised VBM analysis pipeline, and we employed a sensitive statistical methodology: permutation-based cluster statistics. Test statistics for image analysis that incorporate spatial neighbourhood information, such as 3D cluster size, are generally more powerful than test statistics that are informed only by data at a single voxel. There are also disadvantages of our study: we considered the Val158Met SNP in isolation whereas previous evidence indicates it operates at a cellular and macroscopic level in interaction with other SNPs [44], [45]. Given the evidence that extracellular dopamine is linearly related to “dose” of Met alleles, we employed a model to examine regions where brain structure related linearly to “dose” of Met alleles. However, it is possible that there may be non-linear effects of dopamine on brain structure: our model would not detect such effects.

We examined only a single SNP rs4680. Indeed, further loci within COMT may also influence brain structure: we now know that several other polymorphisms in partial linkage disequilibrium with Val158Met have major effects on levels of COMT activity [46]. These additional loci, along functional polymorphisms in the COMT promoter regions, may also have important effects on brain structure [45] and function [47]. Future studies in much larger samples, could address the effects on brain structure of these other COMT SNPs, and interactions between Val158Met and these and other sources of genetic variation, which may also contribute to the differences in brain morphology observed in this study.

## References

[1] Lachman HM, Papolos DF, Saito T, Yu YM, Szumlanski CL (1996). Human catechol-O-methyltransferase pharmacogenetics: description of a functional polymorphism and its potential application to neuropsychiatric disorders.. Pharmacogenetics.

[2] Yavich L, Forsberg MM, Karayiorgou M, Gogos JA, Mannisto PT (2007). Site-specific role of catechol-O-methyltransferase in dopamine overflow within prefrontal cortex and dorsal striatum.. J Neurosci.

[3] Egan MF, Goldberg TE, Kolachana BS, Callicott JH, Mazzanti CM (2001). Effect of COMT Val108/158 Met genotype on frontal lobe function and risk for schizophrenia.. Proc Natl Acad Sci U S A.

[4] Barnett JH, Scoriels L, Munafo MR (2008). Meta-analysis of the cognitive effects of the catechol-O-methyltransferase gene Val158/108Met polymorphism.. Biol Psychiatry.

[5] Mier D, Kirsch P, Meyer-Lindenberg A (2010). Neural substrates of pleiotropic action of genetic variation in COMT: a meta-analysis.. Mol Psychiatry.

[6] Williams GV, Goldman-Rakic PS (1995). Modulation of memory fields by dopamine D1 receptors in prefrontal cortex.. Nature.

[7] Girault JA, Greengard P (2004). The Neurobiology of Dopamine Signaling.. Archives Neurology.

[8] Neckameyer WS (1996). Multiple roles for dopamine in Drosophila development.. Dev Biol.

[9] Dorph-Petersen KA, Pierri JN, Perel JM, Sun Z, Sampson AR (2005). The influence of chronic exposure to antipsychotic medications on brain size before and after tissue fixation: a comparison of haloperidol and olanzapine in macaque monkeys.. Neuropsychopharmacology.

[10] Chua SE, Cheung C, Cheung V, Tsang JT, Chen EY (2007). Cerebral grey, white matter and csf in never-medicated, first-episode schizophrenia.. Schizophr Res.

[11] McClure RK, Phillips I, Jazayerli R, Barnett A, Coppola R (2006). Regional change in brain morphometry in schizophrenia associated with antipsychotic treatment.. Psychiatry Res.

[12] Thompson PM, Cannon TD, Narr KL, van Erp T, Poutanen VP (2001). Genetic influences on brain structure.. Nat Neurosci.

[13] Toga AW, Thompson PM (2005). Genetics of brain structure and intelligence.. Annu Rev Neurosci.

[14] Wright IC, Sham P, Murray RM, Weinberger DR, Bullmore ET (2002). Genetic contributions to regional variability in human brain structure: methods and preliminary results.. Neuroimage.

[15] Zinkstok J, Schmitz N, van Amelsvoort T, de Win M, van den Brink W (2006). The COMT val158met polymorphism and brain morphometry in healthy young adults.. Neurosci Lett.

[16] Kates WR, Antshel KM, Abdulsabur N, Colgan D, Funke B (2006). A gender-moderated effect of a functional COMT polymorphism on prefrontal brain morphology and function in velo-cardio-facial syndrome (22q11.2 deletion syndrome).. Am J Med Genet B Neuropsychiatr Genet.

[17] Harrison PJ, Tunbridge EM (2008). Catechol-O-methyltransferase (COMT): a gene contributing to sex differences in brain function, and to sexual dimorphism in the predisposition to psychiatric disorders.. Neuropsychopharmacology.

[18] Chen J, Lipska BK, Halim N, Ma QD, Matsumoto M (2004). Functional analysis of genetic variation in catechol-O-methyltransferase (COMT): effects on mRNA, protein, and enzyme activity in postmortem human brain.. Am J Hum Genet.

[19] Gogos JA, Morgan M, Luine V, Santha M, Ogawa S (1998). Catechol-O-methyltransferase-deficient mice exhibit sexually dimorphic changes in catecholamine levels and behavior.. Proc Natl Acad Sci U S A.

[20] Barnett JH, Heron J, Ring SM, Golding J, Goldman D (2007). Gender-specific effects of the catechol-O-methyltransferase Val108/158Met polymorphism on cognitive function in children.. Am J Psychiatry.

[21] Pooley EC, Fineberg N, Harrison PJ (2007). The met(158) allele of catechol-O-methyltransferase (COMT) is associated with obsessive-compulsive disorder in men: case-control study and meta-analysis.. Mol Psychiatry.

[22] Xie T, Ho SL, Ramsden D (1999). Characterization and implications of estrogenic down-regulation of human catechol-O-methyltransferase gene transcription.. Mol Pharmacol.

[23] Worda C, Sator MO, Schneeberger C, Jantschev T, Ferlitsch K (2003). Influence of the catechol-O-methyltransferase (COMT) codon 158 polymorphism on estrogen levels in women.. Hum Reprod.

[24] Rantakallio P (1969). Groups at risk in low birth weight infants and perinatal mortality.. Acta Paediatr Scand.

[25] Tanskanen P, Ridler K, Murray GK, Haapea M, Veijola JM (2010). Morphometric brain abnormalities in schizophrenia in a population-based sample: relationship to duration of illness.. Schizophr Bull.

[26] Murray GK, Veijola J, Moilanen K, Miettunen J, Glahn DC (2006). Infant motor development is associated with adult cognitive categorisation in a longitudinal birth cohort study.. J Child Psychol Psychiatry.

[27] Smith SM (2002). Fast robust automated brain extraction.. Hum Brain Mapp.

[28] Zhang Y, Brady M, Smith S (2001). Segmentation of brain MR images through a hidden Markov random field model and the expectation-maximization algorithm.. IEEE Trans Med Imaging.

[29] Jenkinson M, Bannister P, Brady M, Smith S (2002). Improved optimization for the robust and accurate linear registration and motion correction of brain images.. Neuroimage.

[30] Jenkinson M, Smith S (2001). A global optimisation method for robust affine registration of brain images.. Med Image Anal.

[31] Andersson JL, Jenkinson M, Smith S (2007). Non-linear optimisation. FMRIB, University of Oxford..

[32] Andersson JL, Jenkinson M, SMith S (2007). Non-linear registration, aka Spatial normalisation FMRIB, University of Oxford..

[33] Rueckert D, Sonoda LI, Hayes C, Hill DL, Leach MO (1999). Nonrigid registration using free-form deformations: application to breast MR images.. IEEE Trans Med Imaging.

[34] Honea R, Verchinski BA, Pezawas L, Kolachana BS, Callicott JH (2009). Impact of interacting functional variants in COMT on regional gray matter volume in human brain.. Neuroimage.

[35] Suckling J, Barnes A, Job D, Brenan D, Lymer K (2010). Power calculations for multicenter imaging studies controlled by the false discovery rate.. Hum Brain Mapp.

[36] Suckling J, Barnes A, Job D, Brennan D, Lymer K (2012). The neuro/PsyGRID calibration experiment: Identifying sources of variance and bias in multicenter MRI studies.. Hum Brain Mapp.

[37] Cerasa A, Gioia MC, Labate A, Liguori M, Lanza P (2008). Impact of catechol-O-methyltransferase Val(108/158) Met genotype on hippocampal and prefrontal gray matter volume.. Neuroreport.

[38] Ho BC, Wassink TH, O'Leary DS, Sheffield VC, Andreasen NC (2005). Catechol-O-methyl transferase Val158Met gene polymorphism in schizophrenia: working memory, frontal lobe MRI morphology and frontal cerebral blood flow.. Mol Psychiatry.

[39] Ohnishi T, Hashimoto R, Mori T, Nemoto K, Moriguchi Y (2006). The association between the Val158Met polymorphism of the catechol-O-methyl transferase gene and morphological abnormalities of the brain in chronic schizophrenia.. Brain.

[40] Dutt A, McDonald C, Dempster E, Prata D, Shaikh M (2009). The effect of COMT, BDNF, 5-HTT, NRG1 and DTNBP1 genes on hippocampal and lateral ventricular volume in psychosis.. Psychol Med.

[41] Cerasa A, Cherubini A, Quattrone A, Gioia MC, Tarantino P (2010). Met158 variant of the catechol-O-methyltransferase genotype is associated with thicker cortex in adult brain.. Neuroscience.

[42] Shaw P, Wallace GL, Addington A, Evans A, Rapoport J (2009). Effects of the Val158Met catechol-O-methyltransferase polymorphism on cortical structure in children and adolescents.. Mol Psychiatry.

[43] McIntosh AM, Baig BJ, Hall J, Job D, Whalley HC (2007). Relationship of catechol-O-methyltransferase variants to brain structure and function in a population at high risk of psychosis.. Biol Psychiatry.

[44] Tan HY, Nicodemus KK, Chen Q, Li Z, Brooke JK (2008). Genetic variation in AKT1 is linked to dopamine-associated prefrontal cortical structure and function in humans.. J Clin Invest.

[45] Honea RA, Vidoni E, Harsha A, Burns JM (2009). Impact of APOE on the Healthy Aging Brain: A Voxel-Based MRI and DTI Study.. J Alzheimers Dis.

[46] Nackley AG, Shabalina SA, Tchivileva IE, Satterfield K, Korchynskyi O (2006). Human catechol-O-methyltransferase haplotypes modulate protein expression by altering mRNA secondary structure.. Science.

[47] Barnett JH, Heron J, Goldman D, Jones PB, Xu K (2009). Effects of catechol-O-methyltransferase on normal variation in the cognitive function of children.. Am J Psychiatry.

